# The multifarious role of callose and callose synthase in plant development and environment interactions

**DOI:** 10.3389/fpls.2023.1183402

**Published:** 2023-05-31

**Authors:** Ning Li, Zeng Lin, Peiyao Yu, Yanling Zeng, Shenxiu Du, Li-Jun Huang

**Affiliations:** ^1^ State Key Laboratory of Cultivation and Protection for Non-Wood Forest Trees, College of Forestry, Central South University of Forestry and Technology, Changsha, China; ^2^ Key Laboratory of Forest Bio-resources and Integrated Pest Management for Higher Education in Hunan Province, Central South University of Forestry and Technology, Changsha, China; ^3^ Biotechnology Research Institute, Chinese Academy of Agricultural Sciences, Beijing, China

**Keywords:** callose, glucan-synthase-like, plasmodesmata, cell wall, plant-pathogen interaction

## Abstract

Callose is an important linear form of polysaccharide synthesized in plant cell walls. It is mainly composed of β-1,3-linked glucose residues with rare amount of β-1,6-linked branches. Callose can be detected in almost all plant tissues and are widely involved in various stages of plant growth and development. Callose is accumulated on plant cell plates, microspores, sieve plates, and plasmodesmata in cell walls and is inducible upon heavy metal treatment, pathogen invasion, and mechanical wounding. Callose in plant cells is synthesized by callose synthases located on the cell membrane. The chemical composition of callose and the components of callose synthases were once controversial until the application of molecular biology and genetics in the model plant *Arabidopsis thaliana* that led to the cloning of genes encoding synthases responsible for callose biosynthesis. This minireview summarizes the research progress of plant callose and its synthetizing enzymes in recent years to illustrate the important and versatile role of callose in plant life activities.

## 1 Introduction

As early as more than 100 years ago, callose was first found in the phloem sieve plates, microspore mother cells and pollen tubes of plants by staining with the callose specific dye aniline blue (Kauss, 1996); however, it was not until half century later that the chemical composition and molecular structure of callose was characterized (Aspinall and Kessler, 1957). Subsequently, more evidence indicated that, unlike cellulose (β-1,4-glucan), the main component of plant cell walls, callose is a chain of β-1,3-glucan and contains a small amount of β-glucan-1,6-linkages (Meikle et al., 1991). For a long time, scientists tried to purify callose synthase through biochemical means. Paradoxically, the purified protein complex has weak cellulose synthase activity, the molecular nature of plant callose synthase remain speculative and controversial (Verma and Hong, 2001). The successful application of genetics and molecular biology in the model plant *Arabidopsis thaliana* has made a series of important progress in the study of callose synthase, 12 members of the callose synthase family were cloned and characterized in the Arabidopsis genome. The physiological function and biochemical mechanism of callose synthase were elucidated. Members of the callose synthase family are specifically expressed in different plant tissues at different stages, or upon pathogen infection, to synthesize callose and participate in the regulation of plant development and physiological functions.

## 2 Chemical structure and biosynthesis of callose

**Figure 1:**
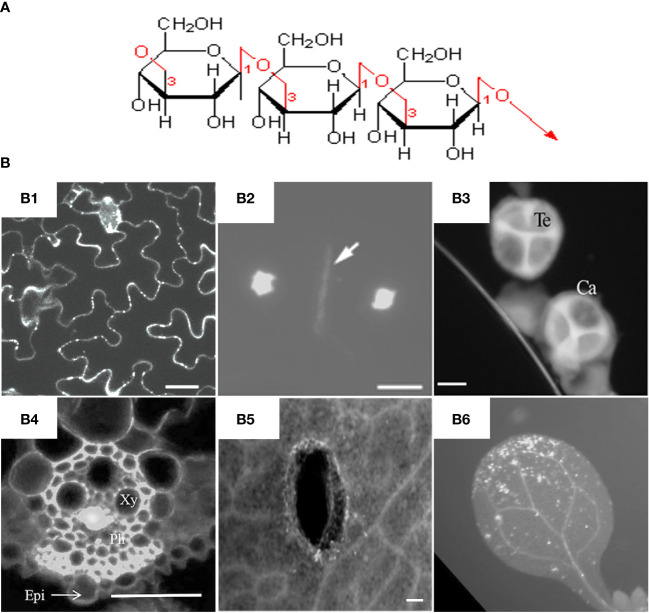
Molecular structure and fluorescent dye staining of callose. **(A)** Callose is composed of a long chain of monomer glucoses joined together by β-1,3-glycosidic bonds. **(B)** Typical images of aniline blue stained callose in different tissues and plant species. (B1) Callose deposition at the plasmodesmata of Arabidopsis leaf epidermal cells, scale bar = 20 µm (Vu et al., 2022). (B2) Callosic cell plate formation (indicated by arrow) during cytokinesis of rice pollen mother cells, scale bar = 10 μm (Zhang et al., 2018). (B3) Callose layer surrounding tetrad microspores in anther of rapeseed, Ca = callose, Te = tetrad, scale bar = 10 µm (Liu et al., 2017). (B4) Virus-induced callose deposition in rice phloem, Xy = xylem, Ph=phloem, Epi = epidermis, scale bar = 50 μm (Yi et al., 2021). (B5) Mechanical wounding-induced callose deposition in cotyledons of rapeseed, scale bar 100 μm, (Liu et al., 2018). (B6) Flg22-induced callose accumulation in Arabidopsis leaves (Keppler et al., 2018). Frames B1-B6 were reproduced with permission under the Creative Commons (CC) licenses.

In general, callose is a glucan chain composed of a raft of glucose residues via β-1,3-glycosidic bonds (**Figure 1A**). In different types of cell walls, the structure and chemical composition of callose may be slightly different, for example, the alcoholic hydroxyl group on the glucose residue ring can be modified by esterification (Chen and Kim, 2009). While as cellulose is mainly composed of polysaccharide chains formed by glucose residues through β-1,4-glycosidic bonds (Ellinger et al., 2014). Unlike cellulose, which has a crystalline structure, callose forms rather a helical or amorphous structure without clearly defined shape.

Callose is mainly synthesized during the specific period of cell wall formation, and is an important component of plant vascular bundles, microspore outer walls, and dividing cell plates (**Figure 1B**). The synthesis and degradation of callose at the plasmodesmata in the cell wall is a major way to regulate the transport of substances between symplasts. Callose is also involved in the formation of phloem sieve plates. Callose accumulates on the sieve plates and controls the pore size to regulate the transport of substances in the phloem (Barratt et al., 2011; Xie et al., 2011). It was found recently that the dynamic accumulation of callose plays an important role in regulating the bud dormancy process of perennial plants (Singh et al., 2018; Tylewicz et al., 2018; Singh et al., 2019). Under short-day conditions, abscisic acid (ABA) led to increased callose synthesis in poplar shoot tips, thereby limiting the activity of shoot tip meristems and suspending growth to avoid freezing damage. Callose plays an important role in the transmission of information between plant cells and regulates the growth and development of plants at different stages.

Both biotic and abiotic stresses in the environment can induce plant callose synthesis. Measuring of callose accumulation in plant leaves or roots by aniline blue staining has become an important tool for detection microbial diseases or heavy metal toxicity in plants (Ellinger and Voigt, 2014). Callose is considered to be the first physical defense line of plants against environmental stress. Formation of callose at different developmental stages, in different tissue parts, and by various stresses is synthesized by callose synthase located on the cell membrane. The amount of callose in plants is jointly determined by callose synthase (CalS, also known as GSL for glucan-synthase-like) and β-1,3-glucanase (BG), the former is responsible for biosynthesis and the latter is responsible for degradation. β-1,3-glucanase is endonuclease that catalyzes the hydrolysis of β-1,3-glucosidic linkages to produce oligosaccharides with a few of glucose units and glucose monomers. Compared with synthesis, the degradation of callose is relatively less studied.

## 3 Expression and regulation of callose synthases

### 3.1 Callose synthases in model plants

Callose is synthesized by a multi-subunit protein complex on the cell membrane. The most critical catalytic subunit is callose synthase. In the model plant *Arabidopsis thaliana*, 12 callose synthase genes were identified (Verma and Hong, 2001). With the exception of *GSL1* and *GSL5*, the other *GSL* genes contain up to 50 exons. Callose synthase is composed of about 2,000 amino acids and is almost the protein with the largest molecular weight in plants, making them remarkably difficult for biochemical analysis.

Callose synthase is a large transmembrane protein with 14~16 hydrophobic transmembrane domains at the N-terminus and C-terminus of the protein, anchoring the synthase on the plasma membrane. The hydrophilic domain in the middle of the protein is located in the cytoplasm and is the main site of callose synthesis. Other major components attached to the synthase complex include sucrose synthase and UDP-glucose transferase, which provide substrates for callose synthesis (Albrecht and Mustroph, 2003). Glucosyltransferases also recruit Rop1 (Rho-related protein from plant) via protein-protein interaction. Rop1 regulates the synthesis of callose by controlling the activity of glucosyltransferase (Hong et al., 2001b). The callose synthase complex also contains annexin, which regulates the activity of the synthase complex through the concentration of calcium ions (Kauss and Jeblick, 1991). With the progress of scientific research, new components of callose synthase have been discovered, and the composition of the synthase complex has also changed according to different subcellular locations and sites of callose synthesis. High-resolution atomic force microscopy was applied to elucidate the structure of the callose synthase complex in living plants (Wu et al., 2006).

Members of the callose synthase gene family are differently expressed during developmental stages of various plant tissues, and respond specifically to various biotic and abiotic stimuli. The Arabidopsis *GSL1*, *2*, *5*, *8* and *10* are involved in the synthesis of callose during pollen development (Dong et al., 2005; Enns et al., 2005; Nishikawa et al., 2005; Huang et al., 2009; Huang et al., 2013); Both *GSL6* and *GSL8* play a role in cell plate formation during mitosis (Hong et al., 2001a; Chen et al., 2009; Saatian et al., 2018). The callose around the plasmodesmata is mainly synthesized by GSL8 and GSL12, while the callose on the phloem sieve tubes is synthesized by GSL7 (Barratt et al., 2011). GSL8 can also regulate the development and formation of leaf stomata by controlling the transport of substances between leaf cells (Guseman et al., 2010). A low-calcium condition induces the expression of *GSL10* gene in leaves that increases callose accumulation and confers plants resist cell death caused by low calcium (Shikanai et al., 2020).

Purified fungal extracts such as chitin and bacterial flagellin peptide can activate the expression and activity of *GSL5* which induces callose accumulation (Luna et al., 2011). Although these inducers can also stimulate the expression of *GSL6* and *GSL11*, so far there is no evidence that these two genes are involved in pathogen-induced callose synthesis. Therefore, callose synthases can be divided into two groups based on their biological functions, the first group including GSL1, 2, 6, 8 and 10, which are mainly responsible for the synthesis of callose during pollen development and cell division; the second group including GSL3, 4, 5, 7 and 12, which are mainly responsible for the synthesis of callose on the cell wall to defend phytopathogen infection. As for the functions of other members such as GSL9 and 11, further studies are required.

### 3.2 Regulation of callose synthase activity

It was once believed that callose and cellulose were synthesized by the same enzyme, and the final synthesis of callose or cellulose was specifically regulated by calcium concentration or phosphorylation level of the enzyme; further genetics and biochemical studies revealed that the two polysaccharides were biosynthesized by two different enzymes: cellulose synthase and callose synthase (Henrissat and Davies, 2000). Callose synthase protein is synthesized on the endoplasmic reticulum and then transported to the cell membrane through the trans-Golgi apparatus, therefore actin fibers are likely to participate in the distribution of callose synthase in the cell (Han et al., 2019).

Calcium ions (Ca^2+^) play an important role in regulating callose synthesis in plants (Waldmann et al., 1988). However, merely increasing intracellular calcium is not sufficient to promote callose production. *In vitro* biochemical studies have shown that certain positively charged substances (such as polyamines and chitosan) and amphiphilic molecules (such as digitonin and phospholipids) can increase the activity of callose synthase; while some ion chelators, lanthanum ions and unsaturated fatty acids can inhibit callose synthase (Kauss and Jeblick, 1991). High-throughput proteomics analysis identified phosphorylated GSL5 protein fragment, suggesting that the activity of callose synthase can also be regulated by phosphorylation at post-translational level (Kline et al., 2010). Biochemical experiments showed that the receptor kinase CRK2 (Cys-rich receptor-like kinase) interacts with and phosphorylates GSL6, which leads to increased callose synthesis (Hunter et al., 2019).

## 4 Diverse role of callose function in plant development and defense

### 4.1 Callose and plant anther development

Callose plays an important role in the development and maturation of stamens and anthers in plant sexual reproduction. The anther primordia differentiated to form an anther structure with four independent anther cells. Each anther cell is composed of epidermis, fibrous layer, middle layer and tapetum wrapping microsporocyte. Callose begins to synthesize near the tapetum when the microspore mother cell undergoes meiosis. When meiosis is completed, four haploid microspores are isolated by the callose layer, forming a tetrad structure (tetrad). During the maturation process of microspores to pollen, callose is gradually degraded, the anther cell is continuously enlarged, and finally the cell is ruptured to release mature pollen (male gamete). Pollen outer cover pollen wall, including pollen outer wall (exine) and inner wall (intine). The composition and structure of the pollen wall change with the development of the pollen, which plays a protective role in the pollen cytoplasm. As previously mentioned, GSL2 is responsible for the synthesis of callose in the Arabidopsis tapetum and outer pollen wall (Dong et al., 2005; Nishikawa et al., 2005), and the callose in the inner wall is jointly synthesized by GSL1 and GSL5 (Enns et al., 2005). Mutants with loss of *GSL2* gene function cannot form normal pollen exine, resulting in reduced activity of male gametes. GSL8 and GSL10 play a role in the mitosis of microspores. *GSL10* loss-of-function mutants have normal tetrads, but microspore mitosis is disordered and normal pollen cannot be formed, resulting in male sterility and the inability to form homozygotes (Huang et al., 2009). Although the microspores of the *gsl8* mutant have disordered mitosis and a reduced proportion of normal pollen, they can still pollinate and form homozygotes (Töller et al., 2008; Chen et al., 2009). The role of callose in the development of female gametes remains to be studied. Recently, through genetic approach two groups independently reported that *OsGSL5* was responsible for callose deposition in anther locules; *OsGSL5* gene mutation resulted in anthers with less callose deposition, aberrant pollen mother cells and abnormal microspores (Shi et al., 2015; Somashekar et al., 2023). Global transcriptome analysis showed that expression of *OsGSL5* was downregulated in the *Osspl* (*OsSPOROCYTELESS*) mutant which was defective in meiosis-specific callose deposition (Ren et al., 2018). Whereas whether the transcription factor OsSPL directly regulates *OsGSL5* gene expression in rice requires further study, Li et al. reported that the transcription factor GhWRKY15 could bind to and repress *GhCalS4* and *GhCalS8* gene expression in cotton (Li et al., 2020).

### 4.2 Callose and plasmodesmata conductivity

**Figure 2:**
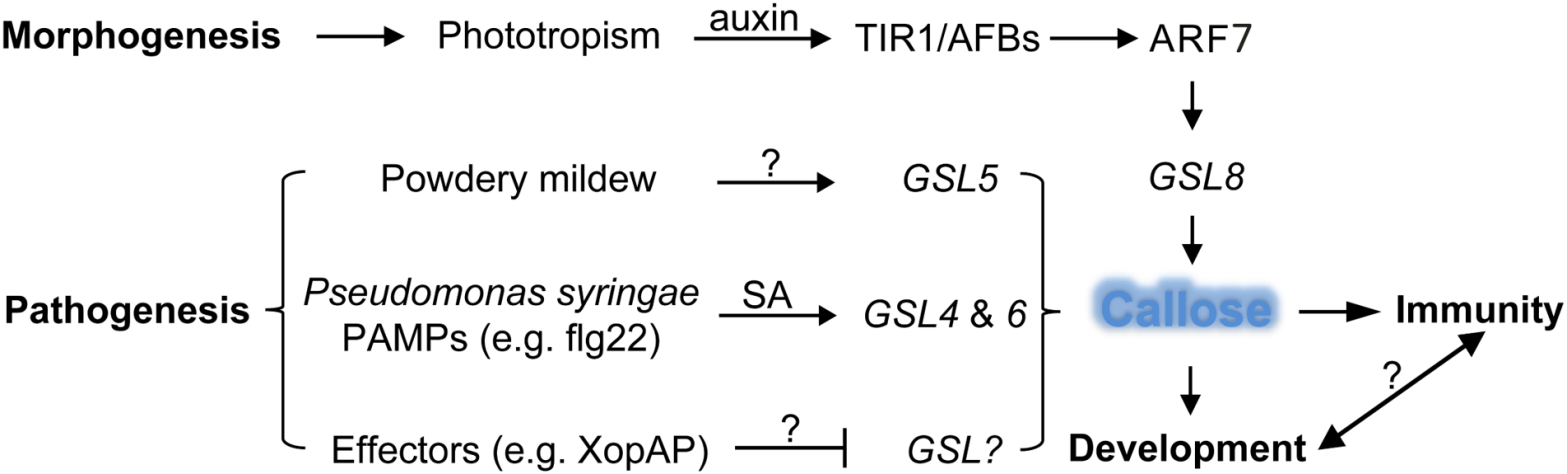
Defense and developmental pathways with respective GSLs to regulate callose synthesis in plants. The morphogen-like plant-growth regulator auxin induces *GSL8* expression through the transcriptional activator ARF7 which is released by the TIR1/AFBs auxin co-receptors to increase callose deposition and mediates plant developmental process, whereas pathogen-induced callose deposition through the activity of GSLs leads to enhanced defense response, so far the signaling crosstalk between development- and immunity-regulated callose deposition is unclear. ARF, auxin response factor; GSL, glucan synthase like; PAMP, pathogen associated molecular pattern; SA, salicylic acid; lines with arrow (↑) indicate positive effect and blunt end (T) negative, question marks (?) denote unknown genes or factors.

The plasmodesmata is a unique structure in plant cells. The plasmodesmata run through the cell wall and are special channels for the transport of material and information symplasts between adjacent cells separated by the cell walls. Similar structures in animal cells are gap junctions. The plasmodesmata are tubular structures formed across the cell wall after the endoplasmic reticulum contracts across the cell plate during mitosis in plant cells. The plasmodesmata allow the passage of proteins, mRNAs, small RNAs (miRNA and siRNA) and viral genomes, and plants selectively transport substances by controlling the permeability. Deposition of callose around plasmodesmata is the main mechanism controlling plasmodesmata pore size. Through reverse genetic screening of 12 members of the Arabidopsis callose synthase family, Han et al. found that *GSL8* is responsible for callose synthesis at the plasmodesmata (Han et al., 2014). Reducing the expression of *GSL8* by RNA silencing technology reduces the callose around the plasmodesmata and causes disruption of the selective transport of auxin. In turn, auxin specifically regulates the expression of *GSL8* gene through the transcription factor ARF7 (**Figure 2**). It is known that auxin can be transported polarly through transport proteins on the cell membrane, but whether auxin is transported directly through plasmodesmata is still unknown. Two independent studies revealed that GSL12 is expressed in the stele and quiescent cells of plant roots and controls root development by regulating the symplast transport of transcription factor SHR and small RNA (miR165) (Vatén et al., 2011; Wu et al., 2016; Yan et al., 2019). It is worth mentioning that transport of small RNAs between plant cells was previously speculated to be through endocytosis and exocytosis; the research on *GSL12* proved directly genetic and cellular evidence that small RNAs can be transported through plasmodesmata.

The sieve pores in the phloem of vascular plants are a special form of plasmodesmata. During the development of the sieve plate, the membrane structure on the plasmodesmata degrades to form the sieve pores. Plants regulate phloem transport by controlling callose deposition around sieve pores. The specific expression of the *GSL7* in vascular tissues is responsible for the deposition of callose in the phloem. *GSL7* loss-of-function mutation leads to the inability of the phloem to transport the carbohydrate nutrients synthesized by leaves (Barratt et al., 2011; Xie et al., 2011).

### 4.3 Callose and plant defense response

Plant viruses spread in plants mainly through plasmodesmata. Virus-encoded movement proteins (MPs), such as the tobacco mosaic virus MP, can change the permeability of plasmodesmata and transport viral genetic material from one cell to another. The dynamic synthesis of callose at plasmodesmata is the major factor that regulates the pore size of plasmodesmata, therefore the spread of plant viruses is largely controlled by callose synthesis. It was found that the plant defense hormone salicylic acid (SA) can induce the biosynthesis of callose near plasmodesmata by activating the expression of *GSL4* and *GSL6* in Arabidopsis, and reduce the intercellular trafficking and restrict virus spreading (Wang et al., 2013; Cui and Lee, 2016) (**Figure 2**). Recently, Huang et al. found that plant Remorin protein-dependent plasma membrane/lipid raft structures are involved in salicylic acid-induced callose synthesis (Huang et al., 2019).

Plant cell walls are the front line of defense against pests and pathogens (Swaminathan et al., 2022). Bacterial infection of plant leaves causes epidermal cells to synthesize a large amount of callose at the invasion site, and the accumulation of callose thickens the cell wall and becomes a physical barrier against bacterial infection. GSL5 (also named PMR4 for POWDERY MILDEW RESISTANT4) is mainly responsible for the synthesis of callose induced by phytopathogen invasion, so as to strengthen the cell wall of the infected or damaged part by pathogens, increase defense, *GSL5* loss-of-function mutants are unable to synthesize callose at sites of bacterial invasion (Vogel and Somerville, 2000; Jacobs et al., 2003; Nishimura et al., 2003) (**Figure 2**). The *GSL* gene family was expanded to 23 members in soybean (*Glycine max*); flg22 treatment upregulated the expression of *GmGSL23*, indicating the essential role of this gene and callose synthesis in the soybean defense response (Sangi et al., 2023). As broached above, not only bacterial infection, but also pathogen-associated molecular patterns (Pathogen-associated molecular patterns, PAMPs) treatment can also induce the activity of callose synthases and the accumulation of callose. The plasma membrane of plasmodesmata is characterized by enrichment of sphingolipids. The t18:0-based sphingolipids specifically facilitate the translocation of glycosylphosphatidylinositol-anchored PDLP5 protein to plasmodesmata and increase accumulation of callose, which lead to elevated resistance to the bacterium *Pseudomonas syringae* and the fungal-wilt pathogen *Verticillium dahlia* (Liu et al., 2020). The beneficial rhizobacteria *Bacillus proteolyticus* OSUB18 and *Bacillus cereus* EC9 triggered induced systemic resistance and callose deposition in host plants to protect against pathogens (Pazarlar et al., 2022; Yang et al., 2023). However, pathogen effectors, such as RxRL3 being secreted by the plant-damaging oomycete *Phytophthora brassicae*, SECP8 by citrus Huanglongbing bacterium *Candidatus* Liberibacter asiaticus and XopAP by rice bacterial blight *Xanthomonas oryzae*, could hamper callose formation (Tomczynska et al., 2020; Liu et al., 2022; Shen et al., 2022). The Avr2-Six5 effector pair of *Fusarium oxysporum* increases plasmodesmal size exclusion limit to facilitate intercellular movement of Avr2 by an unknown mechanism independent of callose deposition (Cao et al., 2018; Blekemolen et al., 2022). Interestingly, some plant-associated beneficial microorganisms, such as *Pseudozyma aphidis*, were able to repress MAMP-elicited callose synthesis to overcome the physical barriers of cell walls and penetrated into plant tissues (Alster et al., 2022). Whether those beneficial microorganisms directly secret glucanases or intervene host immune signaling pathway to reduce callose accumulation still need to be further studied, which will help to understand how plant differentiates endophytic and pathogenic colonization. Aphid infection can lead to callose synthesis. Herbivorous aphids penetrate the phloem through their stylets (needle-like mouthparts) to suck plant juice, and plants block the phloem by accumulating callose on the sieve plates (Will et al., 2013; Jaouannet et al., 2014). The *GhCalS5* gene in cotton (*Gossypium hirsutum*) was induced after aphid feeding and was involved in cotton resistance against aphid attack by mediating callose accumulation (Mbiza et al., 2022). Again, a potential effector NlG14 of the brown planthopper *Nilaparvata lugens* could trigger the accumulation of reactive oxygen species and callose to enhance rice response to both insects and microbe pathogens (Gao et al., 2022; Gao et al., 2023). Recently, Huang et al. deployed the CRISPR/Cas9-mediated gene editing approach to mutate a susceptibility gene in rice, which resulted in mutant lines resistant to root-knot nematode with increased reactive oxygen species burst and enhanced callose deposition (Huang et al., 2023). These findings provide more insights into the molecular mechanisms of plant-pathogen interaction and furnish us with potential targets for the manipulation plant immunity.

### 4.4 The roles of the polysaccharide callose in plant response to metals/metalloids

In addition to biotic stresses like pathogen infection, several abiotic stresses such as heavy metals, could also induce biogenesis of callose *in planta*. The polysaccharide-based biopolymers, such as cellulose, starch and pectin, were proposed as potential biosorption agents for heavy metal removal and environmental remediation (Zhao et al., 2023). It has not escaped the scenario that the carbohydrate polymer callose plays a role in plant-metal/metalloid interactions. So far, interactions of callose and the heavy metal Aluminum and metalloid Silicon are the most well investigated exemplars, which we described in this section. Aluminum is the most abundant metallic element on Earth, accounting for approximately one-tenth of the solid mass on the Earth’s surface. In acidic soils, aluminum ions affect the growth and function of plant roots and are a key factor in slowing plant growth (Ranjan et al., 2021). A growing number of experiments have shown that aluminum treatment leads to callose deposition in plant roots. Through fluorescence staining and electron microscope analysis, it was found that aluminum-induced callose synthesis in wheat roots mainly occurred in plasmodesmata. Further studies revealed that the degree of growth inhibition caused by aluminum was closely related to the decrease in plasmodesmata permeability caused by callose accumulation, which restricts root nutrient and water transport (Bhuja et al., 2004; O’Lexy et al., 2018). Excess heavy metals, such as copper, iron, zinc, and cadmium, inhibit primary root growth by enhancing callose deposition (O’Lexy et al., 2018). Wu et al. found that aluminum-induced accumulation of callose was ameliorated by elevated high pH (Wu et al., 2022a; Wu et al., 2022b). It is still unclear whether heavy metal-induced callose synthesis depends on the phytohormone signaling pathway, and the synthase responsible for aluminum-induced callose synthesis remains to be identified.

Silicon is one of the most abundant elements, accounting for a quarter of the total mass of the crust. Silicon is an essential trace element for the human body, and the silicon element in the human body mainly comes from plant diets. For plants, silicon is not an essential element, and silicon mainly exists in the form of silicon dioxide in plant cell walls (Guerriero et al., 2016). Through the study of the fern *Equisetum arvense*, it was found that plant silicification mainly occurs in callose-rich tissues, such as plant cell walls, cell plates, plasmodesmata (Law and Exley, 2011). *In vitro* biochemical experiments show that in unsaturated Si(OH)_4_ solution, the addition of callose can cause the accumulation of silica. Using silica and callose-specific fluorescent dye analysis, it was found that the accumulation of silica occurred after callose synthesis (Guerriero et al., 2018). Genetic experiments have shown that knockout of callose synthase *GSL5* in the model plant Arabidopsis leads to loss of callose synthesis, which eventually leads to a decrease in silicon content in leaves (Brugiére and Exley, 2017). Similar findings were found in rice (*Oryza sativa* L.), where transgenic rice constitutively expressing a callose hydrolase induced changes in silicon distribution in leaves by degrading callose (Kido et al., 2015). These evidences indicate that callose in plant cell walls is related to silicification. It was hypothesized that the amorphous callose polysaccharides use the hydroxyl groups of their glucosides as a sponge to adsorb and fix granular silicon into the polysaccharide matrix (Exley, 2015; Guerriero et al., 2016). Interestingly, silicon alleviates aluminum toxicity and root growth-inhibition by reducing callose deposition in the cell walls (Jiang et al., 2022; Xiao et al., 2022). It is unclear whether callose can adsorb other chemical elements, although heavy metal ions strongly induce callose synthesis. The role of the cell wall polysaccharide callose in plant response to metals is an emerging new field that deserves more detailed investigation.

## 5 Concluding remarks

**Figure 3:**
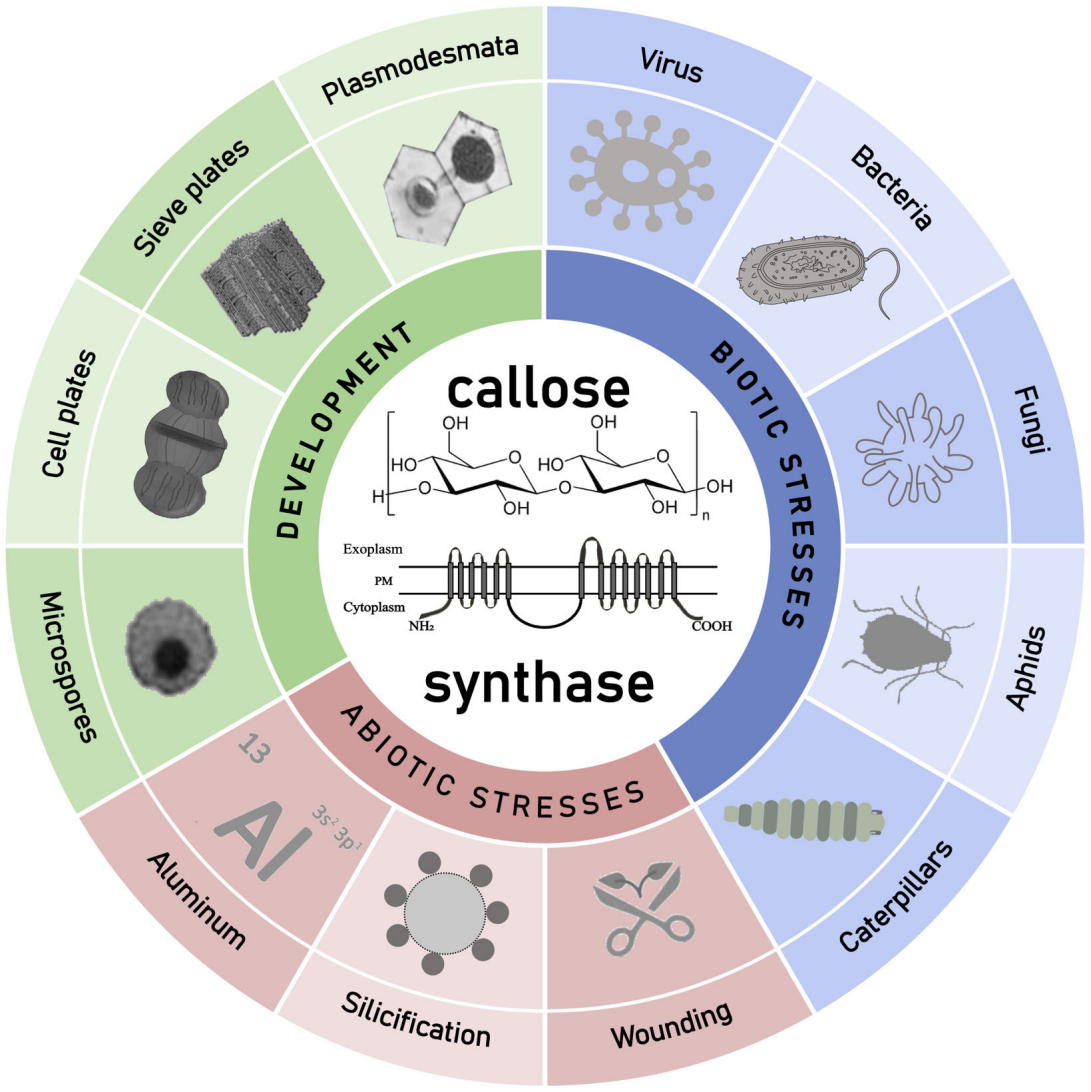
Schematic diagram about the described roles of callose and callose synthases in plant development and environment interaction with emphasis on different kind of pathogenesis. GSLs synthesized callose was found to play multiple roles in plant development, and plant response to various abiotic and biotic stresses.

Callose not only plays an important role in plant development but also participates in plant defense against environmental stresses (**Figure 3**). Recently, progresses have been made in the biological function and biosynthesis of plant callose. Callose synthesized on the cell wall as a cellular barrier against pathogen invasion has attracted much attention. There are still many questions remained, mainly including the following aspects: Are there other components of the callose synthase complex yet to be discovered? Through which signaling pathway is callose synthesis activated during the defense against pathogen invasion? How plants differentiate endophytic friends and pathogenic foes in way of callose induction? Are callose synthases mainly regulated at the transcriptional or post-transcriptional level? Is there a connection between callose and cellulose synthesis or how are they related in the cellular membrane? How to artificially manipulate callose synthesis activity and the callose content in plants? With the development of research techniques, the answers to these key questions will be revealed. The research on plant callose and its biosynthesis is still in the ascendant and has a very promising prospect.

## Author contributions

NL, ZL and L-JH: conceptualization. NL and ZL: literature review. NL and ZL: writing—original preparation. NL, YZ and SD: writing—review and editing. NL, PY, YZ and L-JH: design and revision of the images. All authors contributed to the article and approved the submitted version.
